# Distant hybrids of *Heliocidaris crassispina* (♀) and *Strongylocentrotus intermedius* (♂): identification and mtDNA heteroplasmy analysis

**DOI:** 10.1186/s12862-020-01667-8

**Published:** 2020-08-11

**Authors:** Yaoyao Zhan, Jingxian Sun, Yingying Li, Dongyao Cui, Weijie Zhang, Limeng Yang, Yaqing Chang

**Affiliations:** Key Laboratory of Mariculture & Stock Enhancement in North China’s Sea, Ministry of Agriculture and Rural Affairs, Dalian Ocean University, Dalian, Liaoning 116023 People’s Republic of China

**Keywords:** *Heliocidaris crassispina*, *Strongylocentrotus intermedius*, Distant hybridization, Hybrids identification, MtDNA heteroplasmy

## Abstract

**Background:**

Distant hybridization between the sea urchin *Heliocidaris crassispina* (♀) and the sea urchin *Strongylocentrotus intermedius* (♂) was successfully performed under laboratory conditions. A new variety of hybrid sea urchin (HS hybrid) was obtained. However, the early-development success rates for the HS hybrids were significantly lower than those of purebred *H. crassispina* or *S. intermedius* offspring. In addition, it was difficult to distinguish the HS-hybrid adults from the pure *H. crassispina* adults, which might lead to confusion in subsequent breeding attempts. In this study, we attempted to develop a method to quickly and effectively identify HS hybrids, and to preliminarily investigate the molecular mechanisms underlying the poor early-development success rates in the HS hybrids.

**Results:**

The hybrid sea urchins (HS hybrids) were identified both morphologically and molecularly. There were no significant differences in the test height to test diameter ratios between the HS hybrids and the parents. The number and arrangement of ambulacral pore pairs in the HS hybrids differed from those of the parental lines, which might serve as a useful morphological character for the identification of the HS hybrids. A primer pair that identified the HS hybrids was screened by comparing the mitochondrial genomes of the parental lines. Moreover, paternal leakage induced mitochondrial DNA heteroplasmy in the HS hybrids, which might explain the low rates of early development success in these hybrids.

**Conclusions:**

The distant-hybrid sea urchins were accurately identified using comparative morphological and molecular genetic methods. The first evidence of mtDNA heteroplasmy after the distant hybridization of an echinoderm was also provided.

## Background

Sea urchins are typical inhabitants of shallow coastal areas. Over the past century, sea urchins have been widely used as model organisms in several biological areas, including embryonic development [1], the origins of innate immunity [2], species evolution [3], and marine ecology [4]. Because the human-edible portion of the sea urchin (the gonad) is tasty and nutritious, some sea urchin species are major fishery resources in Asian, Mediterranean, and Western countries [5].

Among the edible sea urchins, the temperate urchin *Strongylocentrotus intermedius* is considered to have the best quality gonads [6]. This species is naturally distributed along the intertidal and subtidal rocky shores of Hokkaido (Japan), the Korean Peninsula, and far eastern Russia [5, 7]. In 1989, *S. intermedius* was introduced to northern China from Japan, and artificial breeding was begun [7]. Currently, *S. intermedius* is the predominant commercially-valuable sea urchin species cultivated along the coast of the north Yellow Sea in China [7]. However, because the thermal tolerance of the species ranges only from − 1 °C to 23 °C [7], the cultivation and promotion of *S. intermedius* in south coastal areas of China have been seriously restricted.

Hybridization has been well documented as a way to create new germplasms and enrich breeding materials [8, 9]. Indeed, crossbreeding has been widely employed to improve growth, survival, stress resistance, and other traits associated with commercial quality in fish [10], shellfish [11], crustaceans [12], and echinoderms [13, 14]. The sea urchin *Heliocidaris crassispina* is naturally distributed along the southeastern coast of China [15]. This species can withstand seawater temperatures as high as 30 °C [16]. However, the market recognition and price of *H. crassispina* is relatively low due to the poor quality of the gonads of this species [7].

**Fig. 1:**
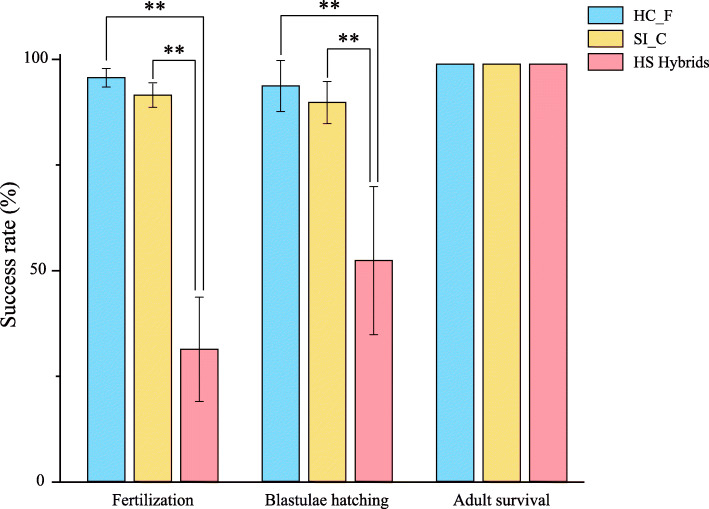
Rate of success at each developmental stage in *Heliocidaris crassispina* purebred offspring, *Strongylocentrotus intermedius* purebred offspring, and *H. crassispina* (♀) × *S. intermedius* (♂) hybrids. HC_F: Purebred offspring of the *H. crassispina* Fujian population; SI_C: Purebred offspring of the *S. intermedius* cultured population; HS: *H. crassispina* (♀) × *S. intermedius* (♂) hybrids. “**” indicates an extremely significant difference (*P* < 0.01) between the two groups connected by the line

**Fig. 2:**
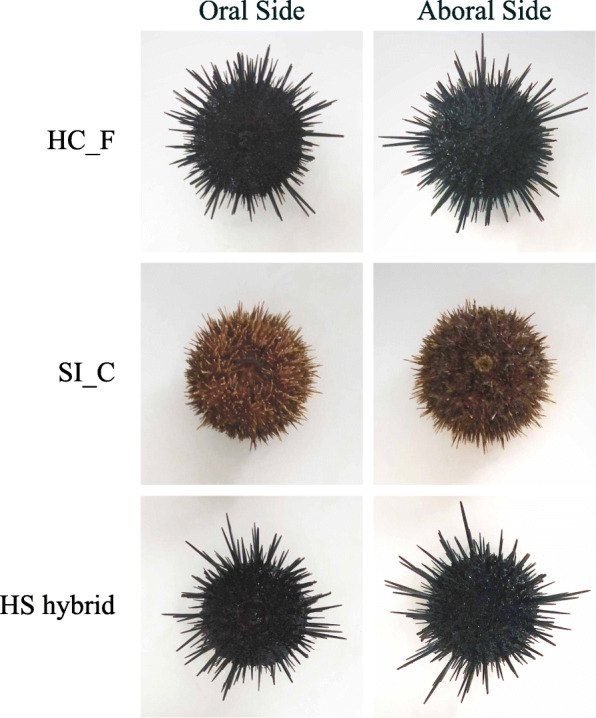
Morphological comparisons among living individuals. HC_F: *Heliocidaris crassispina* Fujian population; SI_C: *Strongylocentrotus intermedius* cultured population; HS hybrid: *H. crassispina* (♀) × *S. intermedius* (♂) hybrids

In an attempt to cultivate a new variety of sea urchin with both high gonad quality and high temperature tolerance, we previously performed a successful distant hybridization between *H. crassispina* (♀) and *S. intermedius* (♂) in our laboratory using a temperature-controlled method [9]. In this method, we spawned and collected gametes at 26 °C (*H. crassispina* ♀) and 21 °C (*S. intermedius* ♂); performed inseminations at 26 °C; and allowed the fertilized eggs to grow and develop at 24 °C [9]. As expected, the larvae produced by the *H. crassispina* (♀) and *S. intermedius* (♂) hybrids (HS hybrids) exhibited increased tolerance of high temperatures, as compared to *S. intermedius* [9]. However, the early-development success rates for the HS hybrids were significantly lower than those of purebred *H. crassispina* or *S. intermedius* offspring (Fig. 1), which might increase the costs of artificial reproduction. Moreover, we found that it was difficult to distinguish the HS-hybrid adults from the pure species *H. crassispina* adults (Fig. 2); this might lead to confusion in subsequent breeding attempts. Thus, effective methods for HS hybrid identification, as well as investigations of the molecular mechanisms underlying the low early development success rates, are urgently needed to support and promote the cultivation of HS hybrids.

External morphology is the traditional basis for species identification [17, 18]. In sea urchins, test height, test width, and the ratio of test height to test width have been used to discriminate groups of wild *H. crassispina* in the South China sea [19]. In addition, the ratio of test width to test length and the ratio of test height to test length were shown to be strongly associated with a geographic location in the sea urchin *Hemicentrotus pulcherrimus* [20]. The number of ambulacral pore pairs is another key character used for morphological classification in sea urchins [21]: Margit [21] divided the family *Strongylocentrotidae* into an oligopore group and a polypore group based on the number of ambulacral pore pairs. It has also been suggested that the developmental trajectories of the arrangement of ambulacral pore pairs were a key character supporting heterochronic evolution in the late Cretaceous echinoid genus *Gauthieria* [22].

Mitochondrial DNA (mtDNA) sequences, which comprise a small portion of the total DNA in a eukaryotic cell, are double-stranded and circular; in most species, mtDNA is inherited solely from the mother [23]. mtDNA sequences are typically 15–20 kb in length, encoding two ribosomal RNA (rRNA) genes, 22 transfer RNA (tRNA) genes, and 13 protein-coding genes [24]. Compared with nuclear DNA, mtDNA has very low levels of recombination and is likely conserved, but mtDNA sequences evolve rapidly [25]. These features render mtDNA sequences useful tools for species identification and the resolution of taxonomic controversies [26, 27]. It has been universally accepted that the mtDNA follows strict maternal inheritance in most animals [28]. However, in recent years, a growing body of evidence has indicated the existence of parental mtDNA transmission and mtDNA heteroplasmy in animals [29]. MtDNA heteroplasmy refers to the existence of more than one mtDNA populations within an individual [29]. In different conditions, mtDNA heteroplasmy has different biological significances. For example, mtDNA heteroplasmy is correlated closely with various disease and aging phenotypes in mice and human beings [30–32], while in amphibians, birds, and arthropods, mtDNA heteroplasmy is considered one of the main factors generating biodiversity [28, 33–35]. In addition, mtDNA heteroplasmy has been identified in several species of fish [36–39]. For example, the delayed elimination of paternal mtDNA might result in a high malformation rate in hybrid yellow catfish during early development [36]. As early as 2000, Steel et al. [40] reported heteroplasmy in the brittlestar *Astrobrach constrictum* and showed that heteroplasmy formed due to mtDNA paternal leakage. However, to our knowledge, this remains the only report of mtDNA paternal leakage and heteroplasmy in echinoderms. As mtDNA heteroplasmy may affect biological processes, species or population diversity, and even evolution, we therefore consider it valuable to broadly assess mtDNA heteroplasmy in as many species as possible.

In the present study, we attempted to develop a method to quickly and effectively identify HS hybrids, and to preliminarily investigate the molecular mechanisms underlying the poor early-development success rates in the HS hybrids. First, external morphological features were analyzed and compared among *H. crassispina*, *S. intermedius,* and the HS hybrids. The complete mitochondrial genomes for *H. crassispina* and *S. intermedius* were then investigated: nucleotide composition and the codon usage profiles of the protein-coding genes (PCGs) were analyzed, and the secondary structure of each identified tRNA gene was described. A molecular primer pair, which can be used to identify living HS hybrids, was designed and validated based on a comparison of the mitochondrial genomes of *H. crassispina* and *S. intermedius*. Lastly, mtDNA heteroplasmy analysis was performed to investigate the molecular mechanisms underlying the low early-development success rates in the HS hybrids.

## Results

### Morphological identification of the HS hybrids

The heights and diameters of the tests of living *Heliocidaris crassispina* (Fujian population) (HC_F), *Strongylocentrotus intermedius* (cultured population) (SI_C), and HS hybrid individuals were measured to calculate the height-diameter ratio for each line. The results indicated that the height-diameter ratio of the HS hybrid was smaller than height-diameter ratios of the parental lines (Table 1), but this difference was not statistically significant (*P* > 0.05).

**Table 1:** Morphological characters of the *Heliocidaris crassispina* Fujian population, the *Strongylocentrotus intermedius* cultured population, and the *H. crassispina* (♀) × *S. intermedius* (♂) hybrids

Sea urchin	Test Height (mm)	Test Diameter (mm)	Test Height / Test Diameter
HC_F	16.11(±1.28)	32.11(±2.14)	0.50
SI_C	14.26(±1.11)	27.89(±1.18)	0.51
HS hybrid	15.30(±1.69)	31.55(±1.24)	0.48

HC_F *H. crassispina* Fujian population, SI_C *S. intermedius* cultured population, HS *H. crassispina* (♀) × *S. intermedius* (♂) hybrids

**Fig. 3:**
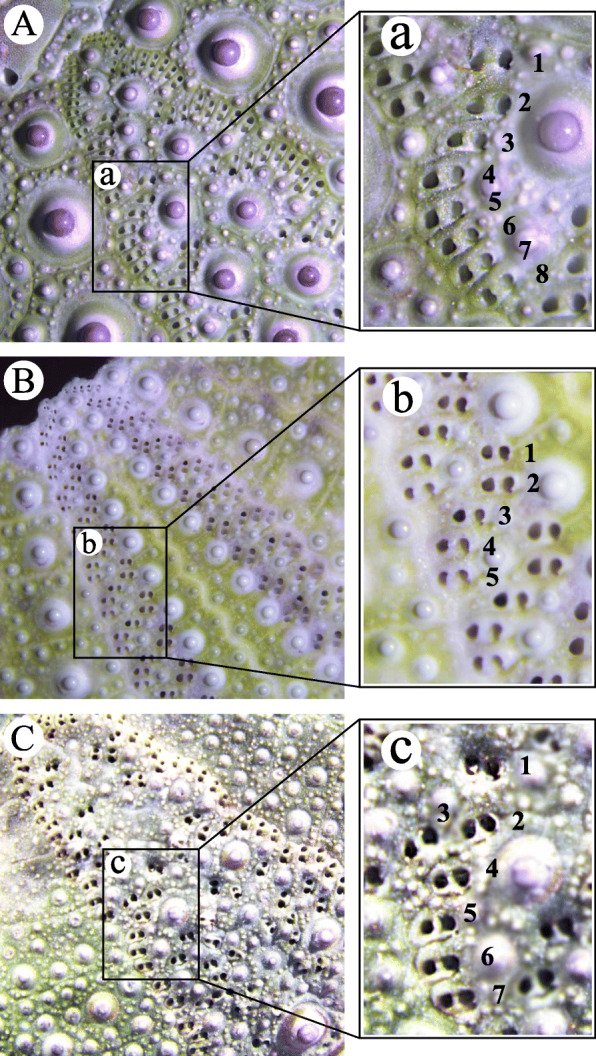
Ambulacral pore pair arrangements and numbers. **a**: Aboral side of test, *Heliocidaris crassispina* Fujian population; **b**: Aboral side of test, *Strongylocentrotus intermedius* cultured population; **c**: Aboral side of test, *H. crassispina* (♀) × *S. intermedius* (♂) hybrid. **a**: Ambulacral pore pairs, *H. crassispina* Fujian population; **b**: Ambulacral pore pairs, *S. intermedius* cultured population; **c**: Ambulacral pore pairs, *H. crassispina* (♀) × *S. intermedius* (♂) hybrids

The tests of sacrificed individuals were observed under a stereomicroscope. Microscope observations revealed six pairs of ambulacral pores in each row on the oral side of HC_F, and seven to nine (mostly eight) pairs of ambulacral pores in each row on the aboral side, which were arranged on the ambulacral plate in an arc shape (Fig. 3). There were five ambulacral pore pairs in each row on both the oral and the aboral side of SI_C, arranged in an oblique arc on the ambulacral plate (Fig. 3). There were four to five ambulacral pore pairs in each row on the oral side of the HS hybrids. These rows were arranged in order but gradually increased at the equator. In each row on the aboral side, we primarily observed seven ambulacral pore pairs. In contrast to the neat arrangement of ambulacral pore pairs in both parents, the ambulacral pore pairs on the aboral side of the HS hybrid were scattered, distorted, and irregular. The numbers and arrangements of the pore pairs were obviously different from those of HC_F (Fig. 3)*.*

### Mitochondrial genomes of HC_F and SI_C

Because mitochondrial genomes may vary among sea urchin species and populations, we designed nine primer pairs based on four reference sequences from the NCBI database (reference sequence accession nos: KC479025.1, KC490912.1, NC_023774.1, and NC_023772.1). From these reference sequences, we developed six primer pairs (I, IV, and VI–IX; Table 2) to amplify the HC_F mitochondrial genome, and five primer pairs (I–V; Table 2) to amplify the SI_C mitochondrial genome. The complete mitochondrial genomes of HC_F and SI_C were cloned and sequenced using polymerase chain reactions (PCRs).

**Table 2:** Primers and reaction conditions for mitochondrial DNA fragment amplification

No.	Primer	Sequence (5′-3′)	HC_F		SI_C		Annealing temperature	Extension time
			Position (bp)	Identity (%)	Position (bp)	Identity (%)		
I	HSM1F	TAACGGATTAAAGCACAGCACTGAA	9879-9903	96.00	9877-9901	100.00	61 °C	3 min 30 s
	HSM1R	CGCATAGAGCTTGAAGGGAATTTAA	13,094-13,118	96.00	13,089-13,113	100.00		
II	HSM2F	TCTTGTTTTCTTGTTTTTGTGAGTT	2812-2836	68.00	12,874-12,898	100.00	54 °C	3 min
	HSM2R	CTCGTGTATCAACATCCATTCC	3541-3562	63.64	886–907	95.45		
III	HSM3F	TGCCATGATTGCAATAGGAGT	825–845	85.71	825–845	100.00	57 °C	3 min 30 s
	HSM3R	ATCTACAAAGTGTCAGTATCAGGCA	4251-4275	92.00	4250-4274	100.00		
IV	HSM4F	CCACTTCTCAACCCATCACCACTTT	4212-4236	92.00	4211-4235	96.00	53 °C	3 min 30 s
	HSM4R	CTATTCCTTGGGGGCCTATTTCTTC	8060-8084	80.00	8058-8082	100.00		
V	HSM5F	TTTTATCTCCTCCCTTTTTATYTCT	8008-8032	76.00	8006-8030	96.00	55 °C	2 min
	HSM5R	CCTCWAAAGTAGTTAAGATTGGGAC	9955-9979	76.00	9953-9977	96.00		
VI	HcM1F	ATCCTGCCTTCCGTTATTTTA	9879-9903	96.00	9877–9901	100.00	53 °C	2 min
	HcM1R	CAACAGTGGTTTGGTCCTTCT	13,094-13,118	96.00	13,089-13,113	100.00		
VII	RandomF	CCGCAAGGGAAAGATGAAATAC	14,289-14,310	100.00	14,285-14,306	100.00	52 °C	4 min
	RandomR	GGGGTGTTATTCTTCTAAGTATTGA	2600-2624	84.00	2598-2622	100.00		
VIII	HcM2F	GAAGGACAAGAACTGGAGAC	2096-2116	100.00	1698-1717	75.00	53 °C	3 min
	HcM2R	CTTTGCGAGATAGATTTAGC	4851–4870	100.00	10,672-10,691	33.33		
IX	HcM3F	ACTTTTGTTTTTCAATAAATCCCTCCA	7680-7706	100.00	8631-8657	70.37	55 °C	2 min 30 s
	HcM3R	TTTCTTTCTAACCACCCTTTTCACC	10,229-10,253	100.00	10,230-10,254	88.00		
X	RDF	TATCATTTAGTAGACCAAAGCCCAT	3559-3583	100.00	3557-3582	100.00	52 °C	40 s
	RDR	CCTGTAGCGACAAAGAAGGTAGAAC	4118-4142	80.00	4117-4141	100.00		

**Fig. 4:**
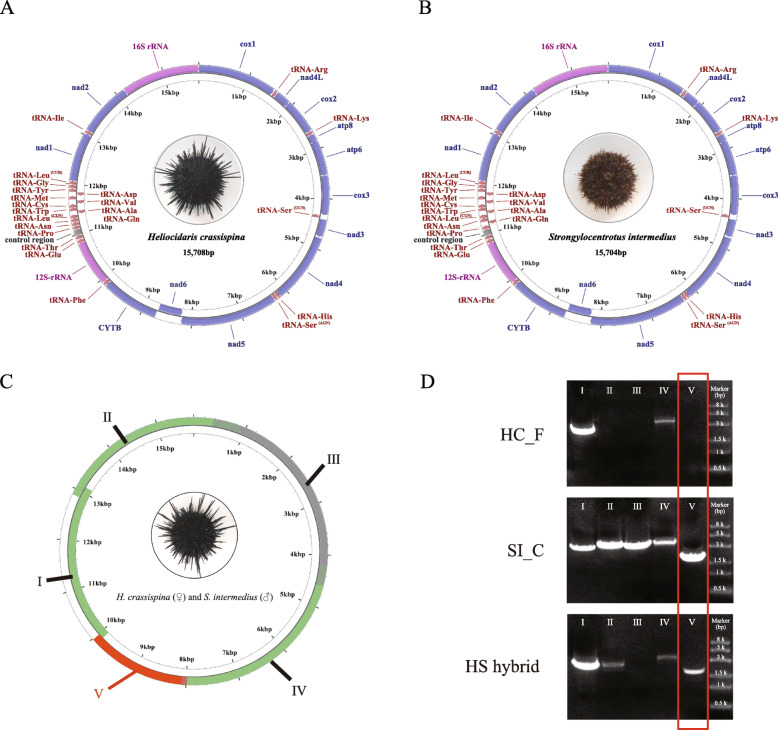
Amplification of mtDNA in *Heliocidaris crassispina* (♀) and *Strongylocentrotus intermedius* (♂) hybrids. **a**: Mitochondrial genome map for the *H. crassispina* Fujian population. **b**: Mitochondrial genome map for the *S. intermedius* cultured population. **c**: mtDNA fragment amplification diagram for the *H. crassispina* (♀) × *S. intermedius* (♂) hybrids. Primer pair V was selected for detection and identification; the product amplified by primer pair V is boxed in red. HC_F: *H. crassispina* Fujian population; SI_C: *S. intermedius* cultured population; HS hybrid: *H. crassispina* (♀) × *S. intermedius* (♂) hybrid. **d**. Electrophoresis gels, showing the results of PCR amplification using primer pairs that were used to amplify mtDNA fragments from the cultured population of *S. intermedius*. The fragments in the inner ring are more similar to the female parent (*H. crassispina*), while the fragments in the outer ring are more similar to the male parent (*S. intermedius*). The PCR products amplified by primer pair V, which was selected for identification, are boxed in red

The mitochondrial genome of HC_F (GenBank accession no. MH899145) was a double-stranded circular DNA with a total length of 15,708 bp and an A + T content of 58.9% (Fig. 4a; Table S1; Table S2). The complete genome contained 37 genes: 13 PCGs, two rRNA genes, and 22 tRNA genes. The largest non-coding region (127 bp) was located between *tRNA*^*Thr*^ and *tRNA*^*Pr*^*°* and was presumably the control region. Together, the protein-coding regions encoded 3822 amino acids (excluding termination codons). The proportion of A + T across the protein-coding regions was 58.2%, and the third codon showed the highest A/U base preference (61.6%). The length of the mitochondrial tRNA was between 68 and 73 bp. With the exception of tRNA^ser^ (AGN), which lacks a dihydrouracil arm, the other secondary structures were typical cloverleaves.

The mitochondrial genome of SI_C (GenBank accession no. MH899146) had a total length of 15,704 bp and an A + T content of 58.9% (Fig. 4b; Table S1; Table S3). Gene number, gene composition, and the location of the putative control region were consistent with the results of the HC_F analysis. The putative control region of SI_C was 125 bp. In total, 3820 amino acids (excluding the stop codon) were encoded by the protein-coding regions, which had an A + T ratio of 58.3%. The third codon also had a strong preference for A/U bases (60.8%). The predicted secondary structure of tRNA^ser^ (AGN) also lacked a dihydrouracil arm.

### Mitochondrial genome comparisons among nine sea urchins

The HC_F mitochondrial genome was 6 bp longer than that of *H. crassispina* (Korean population) (HC_K), as was the HC_F control region. HC_F had single nucleotide polymorphisms (SNPs) at bases 120 and 121 in the control region, in addition to six Gs in the polyguanine portion of the control region. Compared with *S. intermedius* (Korean population) (SI_K), the mitochondrial genome of SI_C was 4 bp longer, while the control region of SI_C was 2 bp longer. SI_C has no base insertions or deletions and no single nucleotide polymorphism except for the two Gs in the polyguanine portion of the control region.

**Fig. 5:**
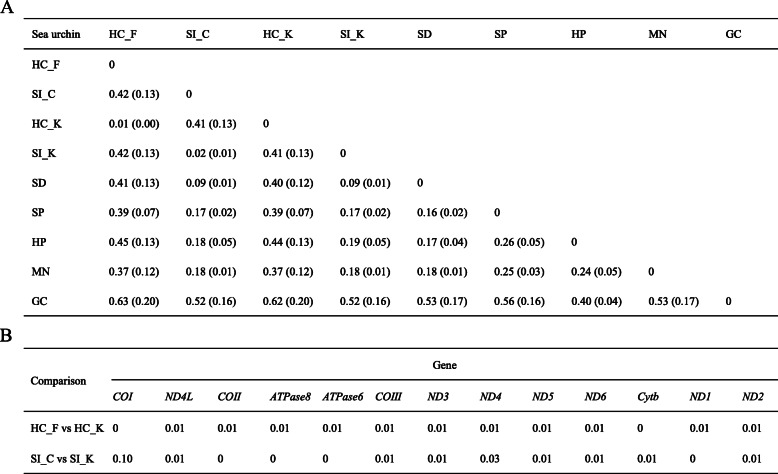
Genetic distance analysis. **a**: Mean distances among common sea urchins for 13 mitochondrial protein-coding genes; standard error is given in brackets. **b**: Genetic distance between the mitochondrial protein-coding genes of different pairs of populations. HC_F: *Heliocidaris crassispina* Fujian population; SI_C: *Strongylocentrotus intermedius* cultured population; HC_K: *H. crassipina* Korean population; SI_K: *S. intermedius* Korean population; SD: *Strongylocentrotus droebachiensis; SP: strongylocentrotus purpuratus*; HP: *Hemicentrotus pulcherrimus*; MN: *Mesocentrotus nudus*; GC: *Glyptocidaris crenularis*

In addition to the populations of *H. crassispina* and *S. intermedius* discussed above, we also selected two widely-distributed and well-studied sea urchin species [*S. droebachiensis* (SD) and *S. purpuratus* (SP)], as well as three common edible sea urchin species from northern China [*H. pulcherrimus* (HP), *Mesocentrotus nudus* (MN) and *Glyptocidaris crenularis* (GC)], for further analysis. The mitochondrial genomes of these nine sea urchin species/populations were compared and analyzed (Table S1). The average genetic distances (K2Ps) among the 13 PCGs in the mitochondrial genomes of HC_F, SI_C, and other common sea urchin species were calculated using MEGA 7 (Fig. 5a). The results indicated that the distance between HC_F and HC_K was 0.01. Compared with the genomes of *Strongylocentrotus purpuratus* and *Cryptocidaris crenularis*, the K2P distances for HC_F and HC_K were identical. When compared to the genomes of other sea urchins, HC_F K2P distances were slightly greater than those of HC_K (0.01). The K2P distance between SI_C and SI_K was 0.02. Compared with *H. pulcherrimus*, the SI_C K2P distances were slightly lower than those of SI_K (0.01). Compared with other sea urchin genomes, the K2P distances for SI_C and SI_K were identical. The genetic distances between HC_F and HC_K and between SI_C and SI_K for the 13 PCGs were calculated using MEGA 7 software (Fig. 5b). The distance between the PCGs of HC_F and HC_K was average, and the differentiation level was low (no more than 0.01). However, *COI* differed noticeably between SI_C and SI_K (0.10); the differentiation in this gene between the two groups was greater than for all other gene pairs (0–0.30).

### Molecular identification of the HS hybrids

Based on the assumption that mtDNA paternal leakage and heteroplasmy occurred in the HS hybrids, we attempted to identify molecular markers using these phenomena that would be suitable for the identification of the HS hybrids. Two sets of PCR amplifications were performed with the five primer pairs used for SI_C mitochondrial genome amplification (primer pairs I–V; Table 2): one set with the total DNA of the HS hybrids as the template, and one set with the total DNA of the HC_F population as the template. As shown in the results, primer pairs I, II, IV, and V were successfully amplified in the HS hybrids, while primer pairs II and V were not successfully amplified in HC_F (Fig. 4d). When these two primer pairs were used, the amplification results differed noticeably. Because the amplification products of primer pair V were shorter than those of primer pair II, the use of primer pair V saves time. Therefore, primer pair V was used for subsequent detection and identification (Fig. 4d).

**Fig. 6:**
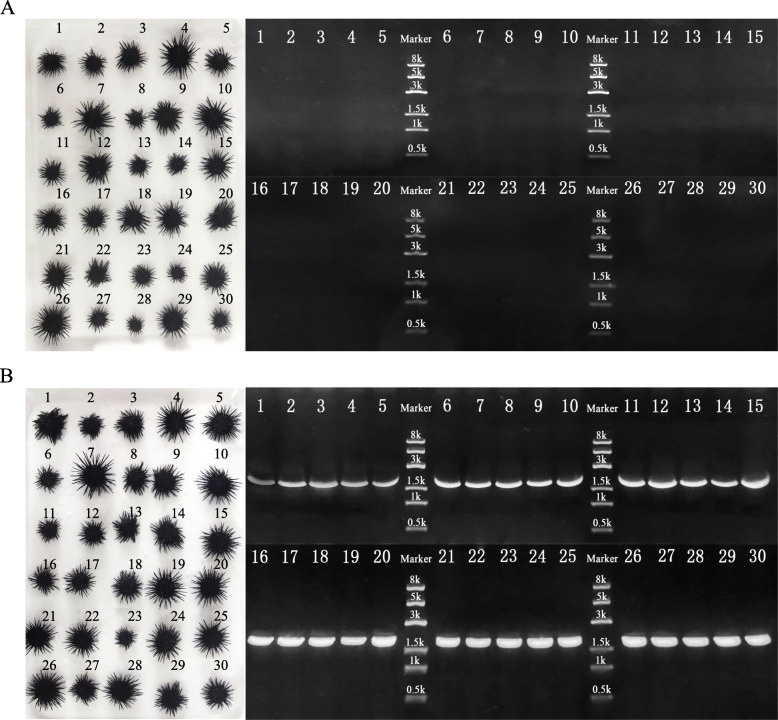
Identification of the *Heliocidaris crassispina* (♀) × *Strongylocentrotus intermedius* (♂) hybrids. **a**: 30 *H. crassispina* from the Fujian population. Electrophoresis gel, showing the PCR products amplified using the identification primer pairs. **b**: 30 *H. crassispina* (♀) × *S. intermedius* (♂) hybrids. Electrophoresis gel, showing the PCR products amplified using the identification primer pair

In order to verify the validity and stability of the primers, we performed PCR amplifications for a large number of samples. Simultaneously, 30 HC_F and HS hybrid individuals were identified. The PCR products were verified using electrophoresis and compared. The electrophoresis results showed that, when primer pair V was used, target bands did not appear in any of the 30 PCRs performed with HC_F total DNA as the template, while target bands did appear for all 30 of the PCRs performed with HS-hybrid total DNA as the template. The latter bands were clear and bright, and the successful identification rate was 100% (Fig. 6).

### MtDNA heteroplasmy in the HS hybrids

**Fig. 7:**
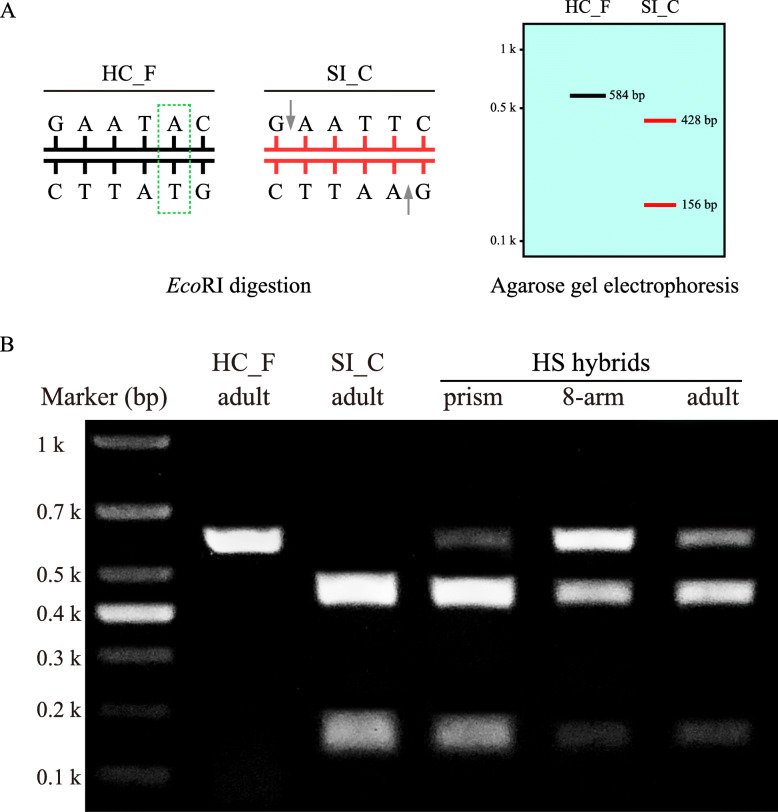
Enzyme digestion of fragments from the parents of the *Heliocidaris crassispina* (♀) × *Strongylocentrotus intermedius* (♂) hybrids and from the pure offspring at each developmental stage. **a**: A 584 bp region was PCR amplified using total DNA as a template. The amplified fragment from the male parent (*S. intermedius*) contains an *EcoR* I-sensitive site. Thus, digestion of this PCR product produces two fragments (428 and 156 bp). However, the amplified fragment from the female parent (*H. crassispina*) is *EcoR* I-resistant. **b**: The fragments amplified from paternal mtDNA can be selectively digested and thus distinguished from the fragments amplified from maternal mtDNA (*H. crassispina*). Lane 1: PCR product resulting from maternal mtDNA amplification. This product could not be digested by the *EcoR* I restriction enzyme and was thus 584 bp long. Lane 2: PCR product resulting from paternal mtDNA amplification was successfully digested by the *EcoR* I restriction enzyme to produce 428 and 156 bp fragments. Lanes 3–5: the biparental mtDNA signal was detected at each stage. HC_F: *H. crassispina* Fujian population; SI_C: *S. intermedius* cultured population; HS hybrids: *H. crassispina* (♀) × *S. intermedius* (♂) hybrid

As paternal mtDNA fragments were amplified from the HS hybrids, we therefore attempted to further verify the occurrence of mtDNA paternal leakage and heteroplasmy in the HS hybrids. After sequencing and multiple sequence alignment, we identified four mtDNA fragments from the HS hybrids that were successfully amplified using primer pairs I, II, IV, and V. We found that sequence I from the HS hybrids was more similar to maternal HC_F (99.29%), while sequences II, IV, and V from the HS hybrids were more similar to paternal SI_C (94.06, 98.71, and 99.59%, respectively; Fig. 4c). Therefore, we preliminarily speculated that the mitochondrial genome of the HS hybrids contained mitochondrial fragments from both paternal lines. To further confirm the simultaneous presence of biparental mtDNA in mitochondrial genome of the HS hybrids, we used restriction digestion to distinguish the biparental mtDNA. PCRs were performed using the primer pair X (Table 2) that amplifies the same fragment of mtDNA in both parents; comparisons of the PCR products identified differing digestion sites. For example, the *EcoR* I digestion of PCR-amplified SI_C mtDNA should produce two fragments, 428 and 156 bp in length. In contrast, PCR-amplified HC_F resists *EcoR* I and should not be digested (Fig. 7a). After PCR and enzyme digestion, only one HC_F band appeared. The length of the band was between 500 and 700 bp, indicating that the PCR products were not digested. However, SI_C generated two bands: one less than 200 bp, and one between 400 and 500 bp. This indicated that the PCR product had been completely digested. However, in the PCR-amplified and digested HS hybrids, we observed not only digested fragments (i.e., bands at 428 and 156 bp) but also undigested fragments. These results were identical across life stages (embryo, larva, and adult; Fig. 7b).

## Discussion

Morphological size comparisons have been successfully used to identify hybrids in several aquatic animal groups, including carp [41], catfish (*Silurus meridionalis* ♀ and *Silurus soldatovi* ♂) [42], and flounder (*Scophthalmus maximus*♀ and *Platichthys stellatus*♂) [43]. Although a previous study suggested that *Hemicentrotus pulcherrimus* populations from different regions were distinguishable based on test size [20], we found no statistically significant differences in traits associated with test size between the HS hybrids and the parental lines, although the tests of the HS hybrids appeared flatter than those of the parents. Thus, we excluded test size as a basis for HS hybrid identification. Consistent with a previous study [21], which stated that the number of ambulacral pore pairs was a key morphological character differentiating sea urchin species, we found an obvious difference in the number of ambulacral pore pairs between the HS hybrids and the parental lines. This character might thus prove useful for HS hybrid identification during artificial breeding. However, because the sea urchin must be sacrificed and the test must undergo complex treatment before the number of ambulacral pore pairs can be counted, it is difficult to apply this method in practice.

We also attempted to identify HS hybrids by searching for and screening genetic molecular markers. We first identified and characterized the mtDNA genomes of the parental lines (*H. crassispina* and *S. intermedius*). Sequencing and bioinformatics analysis showed that dihydrouracil arms were absent from the predicted secondary structures of tRNA^Ser^ (AGN) in both *H. crassispina* and *S. intermedius.* Similar results were also reported in *Mesocentrotus nudus, H. pulcherrimus,* and *Loxechinus albus* [44–46]. We thus hypothesize that the lack of a dihydrouracil arm in the secondary structure of tRNA^Ser^ (AGN) might be common in sea urchins. We also found that the genetic distance between the *COI* gene of the cultured population of *S. intermedius* (in this study) and the *COI* gene from the natural population of *S. intermedius* in Korea [47] was relatively large (0.100). This result might indicate that there is substantial genetic variation between cultured and natural populations of *S. intermedius*. In addition, this result suggested that the *COI* gene represents a potential molecular marker for the discrimination of cultured and natural populations of *S. intermedius*.

As the HS hybrids are very similar to the maternal parent in appearance, we initially predicted that the HS hybrids would strictly adhere to the maternal genetic inheritance of mtDNA. However, when we amplified fragments of the HS hybrid and maternal parent genomes using the five primer pairs that were used to amplify the SI_C mtDNA fragments, the PCR products were not consistent. We noticed that primer pairs II and V, which were supposed to only amplify fragments from *S. intermedius*, also amplified fragments from the HS hybrids. In particular, the use of primer pair V resulted in clear differences in PCR products between the HS hybrids and the maternal parent. Subsequent experiments confirmed that the primer pair V accurately distinguished the HS hybrids from the maternal parent. This primer pair could thus potentially be used in aquaculture settings to distinguish HS hybrids from the maternal parent. Although Havelka et al. [48] suggested that mtDNA could not reliably distinguish hybrids from the maternal parent, our results indicated the opposite. Therefore, we hypothesize that complex mechanisms might control whether mtDNA differs consistently between the hybrid and the maternal parent. Consequently, additional studies are necessary to determine whether mtDNA can be used as a reliable molecular marker for hybrid identification.

Furthermore, sequencing and analysis of mtDNA fragments indicated that some HS hybrid fragments had a higher homology with the paternal parent than with the maternal parent. mtDNA heteroplasmy analysis subsequently suggested that paternal leakage had induced mtDNA heteroplasmy during hybridization. It has been shown that high rates of malformation and death might be closely correlated with the failure to eliminate paternal mtDNA during embryogenesis [36, 39, 49]. Here, mtDNA heteroplasmy was detected in both fertilized embryos and in blastulae. In addition, fertilization and blastula-hatching rates for the hybrids were extremely low. Thus, we speculated that mtDNA heteroplasmy due to paternal leakage might be a key factor affecting fertilization and hatching post-hybridization. Interestingly, mtDNA heteroplasmy was also detected in adult HS hybrids, but there was no significant difference in survival rates between HS hybrid adults and purebred adults. This suggested that the early developmental stages might be more sensitive to the harmful effects induced by mtDNA heteroplasmy than the late developmental stages. Some studies have indicated that paternal mtDNA might not be efficiently eliminated in interspecies hybrids [36, 39, 50]. Our results were consistent with these previous studies and also indicated that paternal mtDNA might not be eliminated in some distant hybrids. In addition, our work raised questions about the dynamic processes comprising mitochondria uniparental inheritance (MUI). In addition, it remains unclear whether mtDNA intermolecular recombination occurred in the HS hybrids. Thus, future studies should aim to clarify not only the species-specific molecular mechanisms associated with the recognition and elimination of paternal mtDNA in hybrid species, but also the occurrence of mtDNA intermolecular recombination in distant hybrids.

## Conclusions

We accurately identified distant-hybrid sea urchins (HS hybrids) using comparative morphological and molecular genetic methods. Moreover, we provided the first evidence of mtDNA heteroplasmy after the distant hybridization of an echinoderm.

## Methods

### Sea urchins

Adult *H. crassispina* (representing one parental line) were collected from the coastal areas of Zhangzhou City, Fujian Province, China (117°28′36″E, 23°42′51″N), and transported to the Key Laboratory of Mariculture & Stock Enhancement in the Ministry of Agriculture of the North China Sea at Dalian Ocean University, Dalian, China. The *S. intermedius* parents were obtained from a broodstock cultured at the Key Laboratory of Mariculture & Stock Enhancement. All specimens were kept in 1000 L recirculating seawater tanks (30 specimens per tank) at room temperature (5 °C–26 °C) under natural light. Specimens were fed kelp (*Saccharina japonica*).

Using methods previously described [9], three lines were developed in September 2016 for the purposes of this research: *H. crassispina* ♀ × *H. crassispina* ♂ (HC_F); *S. intermedius* ♀ × *S. intermedius* ♂ (SI_C); and the distant hybrid *H. crassispina* ♀ × *S. intermedius* ♂ (HS hybrid) (Table 3). All individuals were cultured under identical conditions for 1 year.

**Table 3:** Conditions for the distant hybridization

Sea urchin	Gamete type	Temperature (°C)		
		Gamete collection	Fertilization	Incubation
HC_F	egg	26 (± 0.5)	26 (± 0.5)	24 (± 0.5)
SI_C	sperm	21 (± 0.5)		

HC_F *Heliocidaris crassispina* Fujian population, SI_C *Strongylocentrotus intermedius* cultured population

### Morphological analyses

Living HC_F, SI_C, and HS hybrid individuals were observed, and morphological measurements (i.e., test height and test diameter) were taken following Luo et al. [19]. Using these measurements, the test height-diameter ratios were calculated. Individuals from all three sea urchin lines were sacrificed, and the spines and test skin were removed using a bristle brush. The numbers and arrangements of the ambulacral pore pairs were observed under a stereomicroscope (LEICA M205 FA, Germany).

### DNA sample preparation

Tube-foot tissues were sampled using sterilized ophthalmic scissors and ground thoroughly in liquid nitrogen for DNA extraction. We used TIANamp Marine DNA Kits (Tiangen Biotech, China) to extract total DNA from sea urchins representing each of the three lines. DNA samples were quality controlled using 1% agarose gel electrophoresis and a SimpliNano (BioChrom, UK) ultra-micro-spectrophotometer.

### Sequencing and mitochondrial genome assembly

As mitochondrial genomes might vary among populations, the complete mitochondrial DNA genomes of *S. intermedius* (SI_K), from Jumunjin, on the East Sea of South Korea (GenBank accession no. KC490912); *H. pulcherrimus* from Tongyeong, on the South Sea of South Korea (GenBank accession no. KC490911.1); and *H. crassispina* (HC_K) from Meamul-do, on the South Sea of South Korea (GenBank accession no. KC479025.1) were downloaded from the NCBI database (https://www.ncbi.nlm.nih.gov/). Similar fragments in these sequences were identified using multiple sequence alignments in DNAMAN (Lynnon Biosoft, USA). Based on these similarities, nine pairs of primers were designed to amplify the complete mitochondrial genomes of HC_F and SI_C (Table 2, I–IX). Primer pairs I and IV are universal primers, and were used to amplify mtDNA fragments from both HC_F and SI_C. Primer pairs II, III, and V were specific to SI_C. Primer pairs VI–IX were used to amplify mtDNA fragments in HC_F only. The fragments that were amplified by these primers partially overlapped.

PCRs were performed using *TAKARA LA Taq* (TaKaRa Bio, Japan) in a 50 μl volume, containing 5 μl of DNA template (~ 100 ng/μl), 2 μl of each primer (10 μM each), 5 μl of 10 × LA PCR buffer II (Mg^2+^ plus), 4 μl of dNTP mixture (2.5 mM), 1 μl of LA Taq polymerase (5 U/μl), and 31 μl of DEPC-Treated water. We used an Eppendorf Mastercycler (Eppendorf, Germany) to run the amplification program. The cycling conditions were as follows: pre-denaturation at 94 °C for 5 min; 35 cycles of denaturation at 94 °C for 30 s, annealing at various temperatures for 30 s, and extension at 72 °C for various durations; and a final termination at 72 °C for 5 min (the specific annealing temperatures and extension times used are shown in Table 2). The amplified products were detected using 1% agarose gel electrophoresis, and target bands were cut from the gel. Target bands were purified and fragments were recovered using SanPrep Column DNA Gel Extraction Kits (Sangon Biotech, China). The recovered fragments were cloned using *pEASY*-T1 Cloning Kits and *Trans*5α Chemically Competent Cells (Transgen Biotech, China). DNA fragments were cloned and plasmid DNA was sequenced by Sangon Biotech Co., Ltd. (Shanghai, China). Fragments from the parents of the HS hybrids were assembled using DNAMAN to obtain circular DNA sequences.

### Mitochondrial genome analysis

We located 13 protein-coding genes (PCGs), two rRNA genes, and one control region using the DOGMA service (http://dogma.ccbb.utexas.edu/). The locations of most of the tRNA genes were identified using tRNAscan-SE Search Sever (http://lowelab.ucsc.edu/tRNAscan-SE/) in default search mode with the echinoderm mitochondrial genetic code. Some of the tRNA gene locations could not be identified by tRNAscan-SE; these genes were located using multiple sequence alignments based on the annotations of existing sequences in the NCBI database. Gene maps of the mitochondrial genomes of HC_F and SI_C were generated using CG view Server (http://stothard.afns.ualberta.ca/cgview_server/index.html). tRNAscan-SE and the ARWEN online service (130.235.46.10/ARWEN/) were used to predict the possible secondary structures of the tRNAs. These secondary structures were visualized using the Forna online service (http://rna.tbi.univie.ac.at/forna/). The mitochondrial genomes of the eight other echinoderm species and populations in the NCBI database (Table S1) were compared with HC_F and SI_C using MEGA 7 [51], and the evolutionary distances among the PCGs were calculated (K2P).

### Identification of molecular markers for HS hybrid identification

PCRs were performed using the total DNA from HC_F, SI_C, and the HS hybrid as templates, and five primer pairs that were used to amplify SI_C mtDNA fragments (Table 2, I–V). The electrophoresis results of the resulting amplicons were compared. A pair of primers (Table 2, primer pair V) that effectively identified the HS hybrids was selected. This primer pair was verified against 30 HC_F individuals and 30 HS-hybrid individuals to assess identification accuracy.

### MtDNA heteroplasmy analysis

To detect differential restriction sites, multiple sequence alignments and restriction analyses were performed using the complete mtDNA sequences of the parental lines in DNAMAN. We designed a pair of primers that amplified the same mtDNA fragment from both the maternal and the paternal mtDNA sequences, but carried different restriction sites (Table 2, primer pair X). Restriction digestion was carried out on the PCR fragment, which allowed the amplified mtDNA fragments from SI_C (but not HC_F) to be cut by the *Eco*R I restriction enzyme.

### Data analysis

All data were expressed as mean ± SD. SPSS 22 (IBM, USA) was used for all statistical analyses. Statistical significance was determined using the Tukey test following one-way ANOVAs. We considered *P* < 0.05 significant, and *P* < 0.01 extremely significant.

## Supplementary information

**Additional file 1: Table S1.** Abbreviations and mitochondrial DNA sequence accession numbers for the sea urchin species used in this study. **Table S2.** Characteristics of the mitochondrial genome of the *Heliocidaris crassispina* Fujian population. **Table S3.** Characteristics of the mitochondrial genome of the *Stongylocrntrorus intermedius* cultured population.

## Data Availability

The datasets used and analysed during the current study available from the corresponding author on reasonable request.

## Abbreviations

mtDNA: Mitochondrial DNA
rRNA: Ribosomal RNA
tRNA: Transfer RNA
PCR: Polymerase Chain Reaction
PCG: Protein-coding gene
MUI: Mitochondria uniparental inheritance
HS hybrids: The *Heliocidris crassispina* (♀) and *Strongylocentrotus intermedius* (♂) hybrids
HC_F: *Heliocidaris crassispina* (Fujian population)
HC_K: *Heliocidaris crassispina* (Korea population)
SI_C: *Strongylocentrotus intermedius* (cultured population)
SI_K: *Strongylocentrotus intermedius* (Korea population)
SD: *Strongylocentrotus droebachiensis*
SP: *Strongylocentrotus purpuratus*
HP: *Hemicentrotus pulcherrimus*
MN: *Mesocentrotus nudus*
GC: *Glyptocidaris crenularis*
