# Supplementary material for: Distant hybrids of Heliocidaris crassispina (♀) and Strongylocentrotus intermedius (♂): identification and mtDNA heteroplasmy analysis
Source: BMC Evol Biol. 2020 Aug 11;20:101. doi: 10.1186/s12862-020-01667-8 (PMC7422570; doi:10.1186/s12862-020-01667-8)
Supplement: Supplementary file 1 — Additional file 1: Table S1. Abbreviations and mitochondrial DNA sequence accession numbers for the sea urchin species used in this study. Table S2. Characteristics of the mitochondrial genome of the Heliocidaris crassispina Fujian population. Table S3. Characteristics of the mitochondrial genome of the Stongylocrntrorus intermedius cultured population. [file 12862_2020_1667_MOESM1_ESM.doc]

**Supplementary Material**

**Table S1 Abbreviations and mitochondrial DNA sequence accession numbers for the sea urchin species used in this study.**

| Abbreviation | Sea urchin | GenBank accession no. | NCBI reference sequence |
| --- | --- | --- | --- |
| HC_F | *Heliocidaris crassispina* (Fujian population) | MH899145 | NC_023774.1 |
| HC_K | *Heliocidaris crassispina* (Korea population) | KC479025.1 |
| SI_C | *Strongylocentrotus intermedius* (cultured population) | MH899146 | NC_023772.1 |
| SI_K | *Strongylocentrotus intermedius*(Korea population) | KC490912.1 |
| SD | *Strongylocentrotus droebachiensis* | EU054306.1 | NC_009940.1 |
| SP | *Strongylocentrotus purpuratus* | X12631.1 | NC_001453.1 |
| HP | *Hemicentrotus pulcherrimus* | KC490911.1 | NC_023771.1 |
| MN | *Mesocentrotus nudus* | JX263663.1 | NC_020771.1 |
| GC | *Glyptocidaris crenularis* | KX638403.1 | NC_032365.1 |

**Table S2 Characteristics of the mitochondrial genome of the *Heliocidaris crassispina* Fujian population.**

| *Gene* | Position | |  | Size | |  | Codon | |  | Strand | Intergenic nucleotides |
| --- | --- | --- | --- | --- | --- | --- | --- | --- | --- | --- | --- |
|  | From | To |  | Nucleotides | Amino acids |  | Start | Stop |  |  |  |
| *CO I* | 1 | 1554 |  | 1554 | 517 |  | ATG | TAG |  | + | 0 |
| *tRNA^Arg^* | 1566 | 1634 |  | 69 |  |  |  |  |  | + | 11 |
| *ND4L* | 1636 | 1929 |  | 294 | 97 |  | ATC | TAA |  | + | 1 |
| *CO II* | 1929 | 2618 |  | 690 | 229 |  | ATG | TAA |  | + | -1 |
| *tRNA^Lys^* | 2623 | 2692 |  | 70 |  |  |  |  |  | + | 4 |
| *ATPase8* | 2693 | 2857 |  | 165 | 54 |  | GTG | TAA |  | + | 0 |
| *ATPase6* | 2848 | 3537 |  | 690 | 229 |  | ATG | TAA |  | + | -10 |
| *CO III* | 3541 | 4323 |  | 783 | 260 |  | ATG | TAA |  | + | 3 |
| *tRNA^Ser^(UCN)* | 4323 | 4392 |  | 70 |  |  |  |  |  | - | -1 |
| *ND3* | 4409 | 4759 |  | 351 | 116 |  | ATG | TAA |  | + | 16 |
| *ND4* | 4779 | 6167 |  | 1389 | 462 |  | ATG | TAG |  | + | 19 |
| *tRNA^His^* | 6158 | 6226 |  | 69 |  |  |  |  |  | + | -10 |
| *tRNA^Ser^(AGN)* | 6227 | 6296 |  | 68 |  |  |  |  |  | + | 0 |
| *ND5* | 6296 | 8212 |  | 1917 | 638 |  | ATG | TAA |  | + | -1 |
| *ND6* | 8204 | 8701 |  | 498 | 165 |  | ATG | TAG |  | - | -9 |
| *Cytb* | 8725 | 9867 |  | 1143 | 380 |  | ATG | TAG |  | + | 23 |
| *tRNA^Phe^* | 9867 | 9939 |  | 73 |  |  |  |  |  | + | -1 |
| *12S rRNA* | 9941 | 10831 |  | 891 |  |  |  |  |  | + | 1 |
| *tRNA^Glu^* | 10832 | 10900 |  | 69 |  |  |  |  |  | + | 0 |
| *tRNA^Thr^* | 10911 | 10983 |  | 73 |  |  |  |  |  | + | 10 |
| *tRNA^Pr^°* | 11111 | 11180 |  | 70 |  |  |  |  |  | + | 127 |
| *tRNA^Gln^* | 11182 | 11253 |  | 72 |  |  |  |  |  | - | 1 |
| *tRNA^Asn^* | 11257 | 11328 |  | 72 |  |  |  |  |  | + | 3 |
| *tRNA^Leu^(CUN)* | 11329 | 11400 |  | 72 |  |  |  |  |  | + | 0 |
| *tRNA^Ala^* | 11400 | 11470 |  | 71 |  |  |  |  |  | - | -1 |
| *tRNA^Trp^* | 11472 | 11540 |  | 69 |  |  |  |  |  | + | 1 |
| *tRNA^Cys^* | 11541 | 11608 |  | 68 |  |  |  |  |  | + | 0 |
| *tRNA^Val^* | 11608 | 11677 |  | 70 |  |  |  |  |  | - | -1 |
| *tRNA^Met^* | 11699 | 11771 |  | 73 |  |  |  |  |  | + | 21 |
| *tRNA^Asp^* | 11773 | 11842 |  | 70 |  |  |  |  |  | - | 1 |
| *tRNA^Tyr^* | 11851 | 11921 |  | 71 |  |  |  |  |  | + | 8 |
| *tRNA^Gly^* | 11928 | 11995 |  | 68 |  |  |  |  |  | + | 6 |
| *tRNA^Leu^(UUR)* | 11996 | 12068 |  | 73 |  |  |  |  |  | + | 0 |
| *ND1* | 12071 | 13042 |  | 972 | 323 |  | ATG | TAA |  | + | 2 |
| *tRNA^Ile^* | 13043 | 13113 |  | 71 |  |  |  |  |  | + | 0 |
| *ND2* | 13114 | 14172 |  | 1059 | 352 |  | ATG | TAG |  | + | 0 |
| *16S rRNA* | 14173 | 15708 |  | 1536 |  |  |  |  |  | + | 0 |

Note: “+” indicates that the gene is encoded on the positive strand; “-” indicates that the gene is encoded on the negative strand.

**Table S3 Characteristics of the mitochondrial genome of the *Stongylocrntrorus intermedius* cultured population.**

| *Gene* | Position | |  | Size | |  | Codon | |  | strand | Intergenic nucleotides |
| --- | --- | --- | --- | --- | --- | --- | --- | --- | --- | --- | --- |
|  | From | To |  | Nucleotides | Amino acids |  | Start | Stop |  |  |  |
| *CO I* | 1 | 1554 |  | 1554 | 517 |  | ATG | TAA |  | + | 0 |
| *tRNA*Arg | 1564 | 1632 |  | 69 |  |  |  |  |  | + | 9 |
| *ND4L* | 1634 | 1927 |  | 294 | 97 |  | ATC | TAA |  | + | 1 |
| *CO II* | 1927 | 2616 |  | 690 | 229 |  | ATG | TAA |  | + | -1 |
| *tRNA*Lys | 2621 | 2691 |  | 71 |  |  |  |  |  | + | 4 |
| *ATPase8* | 2692 | 2856 |  | 165 | 54 |  | GTG | TAA |  | + | 0 |
| *ATPase6* | 2847 | 3536 |  | 690 | 229 |  | ATG | TAA |  | + | -10 |
| *CO III* | 3540 | 4322 |  | 783 | 260 |  | ATG | TAG |  | + | 3 |
| *tRNA*Ser(UCN) | 4322 | 4391 |  | 70 |  |  |  |  |  | - | -1 |
| *ND3* | 4408 | 4758 |  | 351 | 116 |  | ATG | TAA |  | + | 16 |
| *ND4* | 4774 | 6162 |  | 1389 | 462 |  | ATG | TAG |  | + | 15 |
| *tRNA*His | 6153 | 6221 |  | 69 |  |  |  |  |  | + | -10 |
| *tRNA*Ser(AGN) | 6222 | 6291 |  | 68 |  |  |  |  |  | + | 0 |
| *ND5* | 6291 | 8210 |  | 1920 | 639 |  | ATG | TAA |  | + | -1 |
| *ND6* | 8211 | 8699 |  | 489 | 162 |  | ATG | TAG |  | - | 3 |
| *Cytb* | 8723 | 9865 |  | 1143 | 380 |  | ATG | TAG |  | + | 20 |
| *tRNA*Phe | 9865 | 9937 |  | 73 |  |  |  |  |  | + | -1 |
| *12S rRNA* | 9938 | 10831 |  | 849 |  |  |  |  |  | + | 0 |
| *tRNA*Glu | 10832 | 10900 |  | 69 |  |  |  |  |  | + | 0 |
| *tRNA*Thr | 10905 | 10977 |  | 73 |  |  |  |  |  | + | 4 |
| *tRNA*^Pr^° | 11103 | 11171 |  | 69 |  |  |  |  |  | + | 125 |
| *tRNA*Gln | 11174 | 11245 |  | 72 |  |  |  |  |  | - | 2 |
| *tRNA*Asn | 11252 | 11323 |  | 72 |  |  |  |  |  | + | 6 |
| *tRNA*Leu(CUN) | 11324 | 11395 |  | 72 |  |  |  |  |  | + | 0 |
| *tRNA*Ala | 11395 | 11465 |  | 71 |  |  |  |  |  | - | -1 |
| *tRNA*Trp | 11468 | 11536 |  | 69 |  |  |  |  |  | + | 2 |
| *tRNA*Cys | 11537 | 11604 |  | 68 |  |  |  |  |  | + | 0 |
| *tRNA*Val | 11604 | 11673 |  | 70 |  |  |  |  |  | - | -1 |
| *tRNA*Met | 11696 | 11768 |  | 73 |  |  |  |  |  | + | 22 |
| *tRNA*Asp | 11770 | 11839 |  | 70 |  |  |  |  |  | - | 1 |
| *tRNA*Tyr | 11847 | 11917 |  | 71 |  |  |  |  |  | + | 7 |
| *tRNA*Gly | 11922 | 11989 |  | 68 |  |  |  |  |  | + | 4 |
| *tRNA*Leu(UUR) | 11990 | 12062 |  | 73 |  |  |  |  |  | + | 0 |
| *ND1* | 12065 | 13036 |  | 972 | 323 |  | ATG | TAA |  | + | 2 |
| *tRNA*Ile | 13038 | 13108 |  | 71 |  |  |  |  |  | + | 1 |
| *ND2* | 13109 | 14167 |  | 1059 | 352 |  | ATG | TAG |  | + | 0 |
| *16S rRNA* | 14168 | 15704 |  | 1537 |  |  |  |  |  | + | 0 |

Note: “+” indicates that the gene is encoded on the positive strand; “-” indicates that the gene is encoded on the negative strand.
